# Interactions between a Candidate Gene for Migration (*ADCYAP1*), Morphology and Sex Predict Spring Arrival in Blackcap Populations

**DOI:** 10.1371/journal.pone.0144587

**Published:** 2015-12-18

**Authors:** Raeann Mettler, Gernot Segelbacher, H. Martin Schaefer

**Affiliations:** 1 Department of Evolutionary Biology and Animal Ecology, University of Freiburg, Freiburg, Germany; 2 School of Natural Sciences, Black Hills State University, Spearfish, South Dakota, United States of America; 3 Wildlife Ecology and Management, University of Freiburg, Freiburg, Germany; Pennsylvania State University, UNITED STATES

## Abstract

Avian research has begun to reveal associations between candidate genes and migratory behaviors of captive birds, yet few studies utilize genotypic, morphometric, and phenological data from wild individuals. Previous studies have identified an association between *ADCYAP1* polymorphism and autumn migratory behavior (restlessness, or zugunruhe), but little is known about the relationship between *ADCYAP1* and spring migratory behavior. The timing of spring migration and arrival to the breeding ground are phenological traits which could be particularly favorable for establishing territories and acquiring mates, thus important to fitness and reproductive success. Here, we investigated how individual genotypic *ADCYAP1* variation and phenotypic variation (wing length and shape) of blackcaps (*Sylvia atricapilla*) affect spring arrival date across nine natural populations in Europe. We hypothesized that longer alleles should be associated with earlier spring arrival dates and expected the effect on arrival date to be stronger for males as they arrive earlier. However, we found that longer wings were associated with earlier spring arrival to the breeding grounds for females, but not for males. Another female-specific effect indicated an interaction between *ADCYAP1* allele size and wing pointedness on the response of spring arrival: greater allele size had a positive effect on spring arrival date for females with rounder wings, while a negative effect was apparent for females with more pointed wings. Also, female heterozygotes with pointed wing tips arrived significantly earlier than both homozygotes with pointed wings and heterozygotes with round wings. Stable isotope ratios (*δ* ^*2*^ *H*) of a subset of blackcaps captured in Freiburg in 2011 allowed us also to assign individuals to their main overwintering areas in northwest (NW) and southwest (SW) Europe. NW males arrived significantly earlier to the Freiburg breeding site than both SW males and females in 2011. NW females had more pointed wing tips compared to SW females, but no difference in *ADCYAP1* allele size was found between the different migration routes.

## Introduction

Humans are fascinated by the seasonal migration of millions of animals around the globe. Avian migration, in particular, has been important to understand the great variation in migratory traits such as morphology, orientation, distance traveled, phenology, and the effects of global climate change on migratory patterns [1,2]. Environmental stimuli, particularly day length and photoperiod, influence migratory behavior by acting as time keepers or ‘zeitgeber’ to synchronize the endogenous, physiological rhythms of migratory animals which oscillate in daily and seasonal patterns [3]. An internal clock or oscillator likely controls a number of circadian and circannual rhythms that influence the behavior of migratory birds [4]. Much progress has been made toward understanding animals’ response to zeitgeber, yet the molecular mechanisms and genes underlying migratory behavior still remain largely unknown [5].

Several migratory traits are heritable and may undergo evolutionary change in short time periods [6]; for example, selective breeding experiments targeted on migratory traits of blackcaps (*Sylvia atricapilla*) suggest that residency and later onset of migratory activity (zugunruhe) can evolve very rapidly [7,8]. Furthermore, a novel migratory orientation/route in wild blackcaps (i.e. migrating north-westerly to overwinter on the British Isles) has evolved only within approximately the past 50 years [9]. In spring, male blackcaps typically arrive earlier to the breeding grounds than females and NW-migrating males tend to precede both SW-males and SW-females [10], thus being able to establish territories first. While migratory behaviors such as onset/intensity of migration and orientation are seemingly complex traits, many actually appear to have simple inheritance patterns. For example, offspring of parents with different migratory orientations were found to have orientations intermediate to their parents [11].

It has been discussed that traits involved in migration may be linked to a ‘migratory gene package’ [3,5,12] and the mechanisms promoting divergence and/or speciation on the genomic level are likely ‘multifaceted’ [13,14]. Likely components of this package are the genes that control migratory restlessness (zugunruhe) and migratory flight (zugstimmung), the latter possibly an exaptation for migration imposing different selective regimes for long-distance migratory flight vs. normal daily movements [1]. Wing length and pointedness have been shown to positively correlate with migratory distance in bird species [15–18]. Yet the ecomorphological constraints imposed by wing morphology on the breeding grounds (e.g. maneuverability and habitat use) may limit the evolvability of wing morphology as compared to the rapid microevolution of migratory behavior [13,16]. Therefore, disentangling migratory features, and the role of genetic and environmental influence on such traits, remains challenging.

Recent studies have begun to investigate ‘candidate genes’ potentially related to migratory behavior in non-model organisms. In songbirds, several potentially informative candidate genes, such as *AANAT*, *ADCYAP1*, *CKIe*, *CLOCK*, *CREB1*, *CRY1*, *NPAS2*, and *PERIOD2*, have been suggested to be associated with circadian rhythmicity, breeding phenology, and migratoryness [19–25]. For example, greater number of *CLOCK* poly-Q repeats predicted later spring migration dates in two trans-Saharan migrants, tree pipits (*Anthus trivialis*) and nightingales (*Luscinia megarhynchos*), when controlling for sex, age, and wing length effects [24]. Mueller et al. [22] identified an association between *ADCYAP1* genotypes and migratory behavior among two captive populations of blackcaps: birds with longer *ADCYAP1* alleles displayed more migratory restlessness in the autumn. While a significant association was similarly found in a single, captive population of juncos (*Junco hyemalis)*, it has been suggested that variation at this locus does not consistently predict migratory behavior across populations or among passerine species [21,24]. Therefore, it remains contentious whether *ADCYAP1* best predicts migratory behavior across a species’ range or within a single population, if such relationships are found to be more strongly associated with migratory distance or migratory timing, and if the effects are consistent among spring and autumn migration. Extending similar genetic methods to multiple, wild populations spanning the repertoire of a species’ behavior has so far only been based on categorical inference of migratory behavior (i.e. migratory status) averaged over populations [22], yet the link between *ADCYAP1* variation and variation within and among wild populations remains largely unexplored (but see [24,26]). We here investigate (i) whether variation in *ADCYAP1* allele size predicts spring arrival time on an individual- and population-level in natural populations of blackcaps and (ii) whether *ADCYAP1* variation is correlated with wing morphology across the species’ range and also within a single breeding population (Freiburg, Germany) comprised of individuals with different migratory strategies. In this well-studied population, where genetic divergence between sympatric NW- vs. SW- migrants has been documented [17,27], we assess the interaction between spring arrival, *ADCYAP1*, and wing morphology.

Based on the relationships previously found with *ADCYAP1* allele size and migratory/breeding behavior in birds [21,28,29], we first predict wild blackcaps with longer *ADCYAP1* alleles to arrive earlier in spring if this gene is more strongly associated with the timing of individual migratory and/or breeding behavior [21,29]. Second, as populations considered here range from intermediate- to long-distance migrants, we predict that blackcaps with shorter and rounder wings (i.e. those migrating shorter distances) would have earlier spring arrival dates across Europe (and similarly within a given population) due to shorter distances travelled to the breeding grounds [9,10,17]. Third, we hypothesize any genetic or phenotypic effects on spring arrival will be more apparent in male blackcaps compared to females, as males incur higher selection pressures to arrive early on the breeding ground and establish territories.

## Methods

### Ethics Statement

The protocol for handling birds and collecting blood samples at field sites for this study was approved by the Regierungspräsidium Freiburg Referat 3 (Regierungspräsidium Freiburg Referat 35, Veterinärwesen, Lebensmittelüberwachung Bertoldstr. 4379098 Freiburg). To minimize stress, individual birds were handled within less than 10 minutes of capture and released unharmed to their original capture sites. Samples from Freiburg have been collected under field work and animal experiment permits granted by the responsible state environmental offices of Baden-Württemberg (RPT Tierversuch-Nr. 55–8853.17/0). Samples collected in Kefermarkt, Austria were approved from the district Freistadt permit Nr. N10-75-2010. Sampling in Rybachy (Russia) was performed under the license from Kaliningrad Regional Agency for Protection, Reproduction and Use of Animal World and Forests. Trapping and sampling permits in Bulgaria were granted under permit N243/1.03.2010 from Bulgarian Ministry of Environment and Waters.

### Morphological Measurements

**Fig 1:**
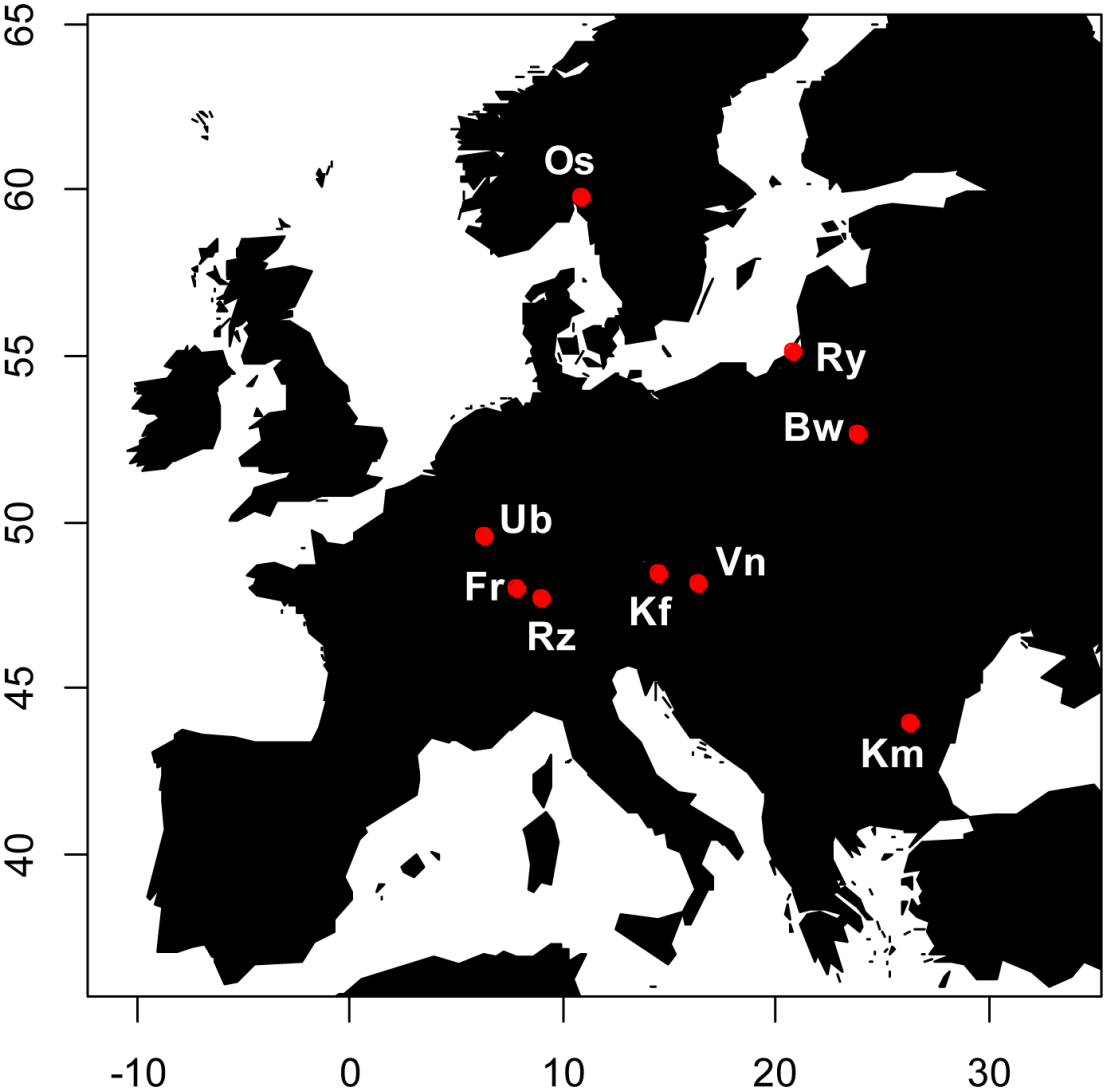
Map of collecting localities in Europe. Population codes: **Ub** = Uebersyren, LU; **Fr** = Freiburg, DE; **Rz** = Radolfzell, DE; **Os** = Oslo, NO; **Kf** = Kefermarkt, AT; **Vn** = Vienna, AT; **Ry** = Rybachy, RU; **Bw** = Białowieża Forest, PL; **Km** = Kalimok, BG. Geographic coordinates for collecting localities can be found in S1 Table. Degrees East and North are shown on the X and Y axes, respectively. Western migrants include: Ub, Fr, Rz, Os; Eastern migrants include: Kf, Vn, Ry, Bw, Km consistent with [27].

A total of 936 adult blackcaps (male *N* = 518; female *N* = 418) were captured upon arrival to the breeding grounds via mist nets at nine locations in Europe across several different years (Fig 1, S1 Table). Collecting sites were monitored daily for first arrivals and equal sampling effort was made across these large breeding populations. Individuals were sampled from 19 March through 31 May (raw dayscore 1–74). Latitude and longitude of collecting locales (S1 Table) should reflect geographic patterns of wing morphology because blackcaps from northern and eastern populations migrate farther than those in the west and south [16]; the former should therefore have longer and more pointed wings [16,30]. Length of the wing chord (hereafter wing length) was measured with a wing chord ruler with a precision of 0.5 mm. The length of primary feathers P1 –P9 and length of the first secondary feather (S1) were measured using a ruler with an attached pin with 0.5 mm precision [31]. Morphological measurements were standardized among ringers by measuring the same individual birds consecutively and adjusting measurements between ringers by the mean of the difference found (e.g. 0.23–0.37 mm correction applied to wing lengths per population); this process was applied for birds captured in Uebersyren, LU (2011), Freiburg, DE (2007–2011), Radolfzell, DE (2006), Kefermarkt, AT (2010, 2011), Vienna, AT (2010), and Białowieża, PL (2011). Blood or feathers were collected from individual blackcaps for genetic analyses and stored at -20°C.

### Lab Methods

The *ADCYAP1* (adenylate cyclase-activating polypeptide 1) gene is a member of the secretin-glucagon growth hormone-releasing hormone (GHRH) / vasoactive intestinal peptide (VIP) superfamily of peptides. This gene has been well conserved throughout vertebrate evolution and encodes a neurotransmitter (*PACAP*, pituitary adenylate cyclase-activating polypeptide) found in the brain and peripheral tissues, where it exhibits pleiotropic effects including several actions on the pineal gland (e.g. secretion of melatonin) and circadian rhythmicity [32–34]. *ADCYAP1* is a polymorphic, dinucleotide microsatellite locus in the 3’ UTR (untranslated region) of avian chromosome 2; at this location are known regulatory elements involved with post-transcriptional processes. For example, the 3’ UTR of *Period3* in mouse modulates mRNA stability and contributes to circadian oscillations of this clock gene [35]. In the chicken pineal gland, in vivo exposure of *PACAP* is known to directly affect the expression of clock genes *CLOCK* and *CRY1*, therefore neurotransmitters may modulate gene expression of the clock components via light in the avian pineal gland [25]. Although the molecular mechanisms responsible for avian circadian rhythmicity and seasonal migration are still largely unknown [4,36], evidence strongly suggests locomotor activity and song/call are controlled by a multioscillatory, central clock system coordinating the interplay between major photoreceptor tissues (retina and pineal), encephalic photoreceptors, and pacemakers (retina, pineal, and suprachiasmatic nuclei) [25,34,37]. Gene-associated tandem repeats, like those found at *ADCYAP1*, have therefore been recommended for investigating genotype-phenotype associations involved with avian circadian and circannual behaviors [20].

Total genomic DNA was extracted from blood and feather samples with Qiagen DNeasy Blood and Tissue Kits. The *ADCYAP1* locus was PCR-amplified in all individuals using published primers [20] with the forward primer labeled with FAM or HEX fluorescent dyes. Reaction volumes of 20μl reactions included: 1x Qiagen CoralLoad Concentrate, 1x Qiagen PCR Buffer (containing KCl and (NH_4_)_2_SO_4_), 0.25 μM labeled forward primer, 0.25 μM reverse primer, 200 μM of each dNTP, 1.25 U Qiagen Top Taq DNA polymerase, and 1.0μl DNA template. Mastercycler Gradient Thermocyclers (Eppendorf GmbH, Hamburg, Germany) were used to run PCR under the following conditions: initial denaturing at 94°C for 5 min, followed by 20 cycles of 94°C for 30 s, 60°C for 30 s (decreased by 0.5°C per cycle), and 72°C for 45 s, followed by 15 cycles of 94°C for 30 s, 50°C for 30 s, and 72°C for 45 s with a final extension of 72°C for 3 min. PCR products were genotyped on a 3130xl Genetic Analyzer (Applied Biosystems), alleles were called with Peak Scanner v1.0 (Applied Biosystems), and binned using Tandem v1.09 [38].

### Data Analysis

Two wing morphology variables were considered: wing length (wing L) and wing pointedness (wing P). Wing lengths were log transformed for statistical analyses. Wing pointedness was calculated from lengths of feathers (P1-P9, S1) and wing lengths using a modified Holynski Index (see [17]), e.g. greater wing pointedness indices correspond with more pointed wing tips. Information for wing pointedness was unavailable for individual blackcaps captured in Oslo (Os) and Kalimok (Km). Raw dayscores (ranging from 1–74) were standardized within populations by setting the mean capture date (S1 Table) for each population to zero and normalizing to positive values (to accommodate later models) by adding the lowest value across all populations to each individual measure. Raw and standardized dayscores were highly correlated (*r* ^*2*^ = 0.598, *p* < 2.2e-16), the latter then served as a proxy for spring arrival date.

The following genetic variables are considered per individual in this study: allele length of the smaller *ADCYAP1* allele (AD1), allele length of the longer *ADCYAP1* allele (AD2), mean allele length at the *ADCYAP1* locus (ADmean), and heterozygosity (0 = homozygous individual, 1 = heterozygous individual, calculated by the ‘rhh’ package in R, [39]). Although some controversy remains about the utility of heterozygosity-fitness correlations (HFCs) in reference to inbreeding, several avian life history traits have indeed been shown to correlate positively with individual heterozygosity, especially across microsatellite markers [40]. Hence we include individual heterozygosity at *ADCYAP1* to study its relationship with spring arrival, yet agree that HFCs are best measured with a large number of markers, e.g. single nucleotide polymorphisms (SNPs) [41]. Kruskal-Wallis rank sum tests were used to characterize sex effects among the morphological and genetic variables considered. Population means were additionally determined for wing morphology measures, *ADCYAP1* allele sizes, and heterozygosity to identify range-wide patterns in this species. Relationships between wing morphology means and latitude/longitude of populations were examined with nested Generalized Linear Models (GLMs, i.e. with longitude nested within latitude) to provide a broad geographic context of phenotypic variation.

To determine if variation in *ADCYAP1* and wing morphology affect spring arrival in blackcaps, GLMs were fit using a negative binomial distribution and log link function with standardized dayscore as the dependent variable and wing morphology (wing L and wing P) and *ADCYAP1* variation (AD1, AD2, ADmean, heterozygosity) each as independent variables in separate analyses. To minimize the potential sample bias from the large number of blackcaps collected in Freiburg, DE (*N* = 441) and test for pattern robustness, five random subsets of 100 males and 100 females were constructed from the total Freiburg population and included in all-population analyses (as Set 1–5). Optimal models were selected based on comparison of the null versus the residual deviance and optimization of the AIC values. In addition to the full data set comprising all nine populations, a morphometrically standardized subset of six populations (Ub, Fr, Rz, Kf, Vn, Bw; Fig 1 and S1 Table) was analyzed to control for wing measurement bias between populations (including random Set 5 from Freiburg). GLMs were estimated using the R package ‘MASS’ [42] and visualized with the ‘effects’ package [43]. To control for multiple comparisons, the Benjamini and Hochberg [44] procedure was used to adjust GLM *p*-values with a 0.10 FDR.

#### Intra-population Data Analysis: Freiburg, DE

An *intra*-population approach was taken to study variation in *ADCYAP1*, wing morphology, and spring arrival in a well-studied population in Freiburg, Germany. As a subset of the total dataset, *N* = 398 (*N*_male_ = 207; *N*_female_ = 191) adult blackcaps were captured upon spring arrival to the breeding grounds in Freiburg, Germany from 2007–2011. Arrival dates of these birds spanned March 19—April 20 over consecutive years in Freiburg. Due to an observed year effect in the overall dataset on spring arrival (GLM *est* = -0.015 ± 0.004, *p* = 0.0003), dayscores were standardized among years based on the calendar date blackcaps were first detected each respective year to control for this effect. Birds were sampled daily for a continuous time frame in the same forest (Freiburg Mooswald) as part of previous studies [10,17,27,45,46]. Arrival day of spring blackcaps in Freiburg was investigated as a response variable to test the effect of wing morphology and *ADCYAP1* variation on arrival date. Males and females were analyzed separately and *p*-values were adjusted using the Benjamini and Hochberg [44] method with FDR = 0.10.

Blackcaps captured in Freiburg in the spring of 2011 (*N* = 98) were analyzed for stable isotopes (*δ* ^*2*^ *H*) in a previous study (from claws in [27]) to discriminate between individuals wintering on SW or NW wintering grounds. A Spearman rank correlation test was conducted to determine the relationship between individual *δ* ^*2*^ *H* isotope and dayscore among these birds. Individuals were further subdivided by migratory direction (NW or SW) and sex (male or female). Wilcox Rank Sum tests were implemented to investigate among-group differences in dayscore, wing length, wing pointedness, and *ADCYAP1* allele size. From observations on the Freiburg breeding ground during 2010–2011 (unpublished data), isotopes from males and females in breeding pairs (total of 23, see [27]) were compared to test for evidence of asssortative mating using a null model by jackknifing (x1000) true breeding females against a pool of 107 males captured in Freiburg in 2010 and 2011; wing morphologies and *ADCYAP1* genotypes were additionally investigated among pairs with similar null models. Relationships between true breeding pairs were compared against the confidence intervals of the normal distributions produced by 1000 random pairs to determine if different from random. All analyses were conducted in R v3.2.1 [47].

## Results

### Genetic variation among all blackcap populations

A total of 11 *ADCYAP1* alleles were found among the nine blackcap populations ranging from 154–174 bp (which are 3bp longer than sizes previously published in [22]); alleles 164 and 168 were most frequent in all populations, with mean frequencies of 0.350 and 0.402, respectively (S2 Table, S1 Fig). Bimodal allele frequencies at *ADCYAP1* (S1 Fig) are consistent with previous studies of this species [22]. Individuals heterozygous at *ADCYAP1* were more numerous and had significantly earlier dayscores (*N* = 655, mean dayscore = 36.72, SE = 0.325) than homozygous individuals (*N* = 281, mean dayscore = 37.92, SE: 0.559; *p* = 0.015; S1 Table). No differences in *ADCYAP1* allele size were found between western and eastern migrants (Wilcoxon rank sum tests: *p* > 0.05 with Set 5; see Fig 1 and [27] for categorization of orientations), while western migrants arrived to breeding grounds earlier than eastern migrants (mean day: 23.82 and 41.39, respectively; Wilcoxon rank sum test: *p* < 2.2e-16).

**Fig 2:**
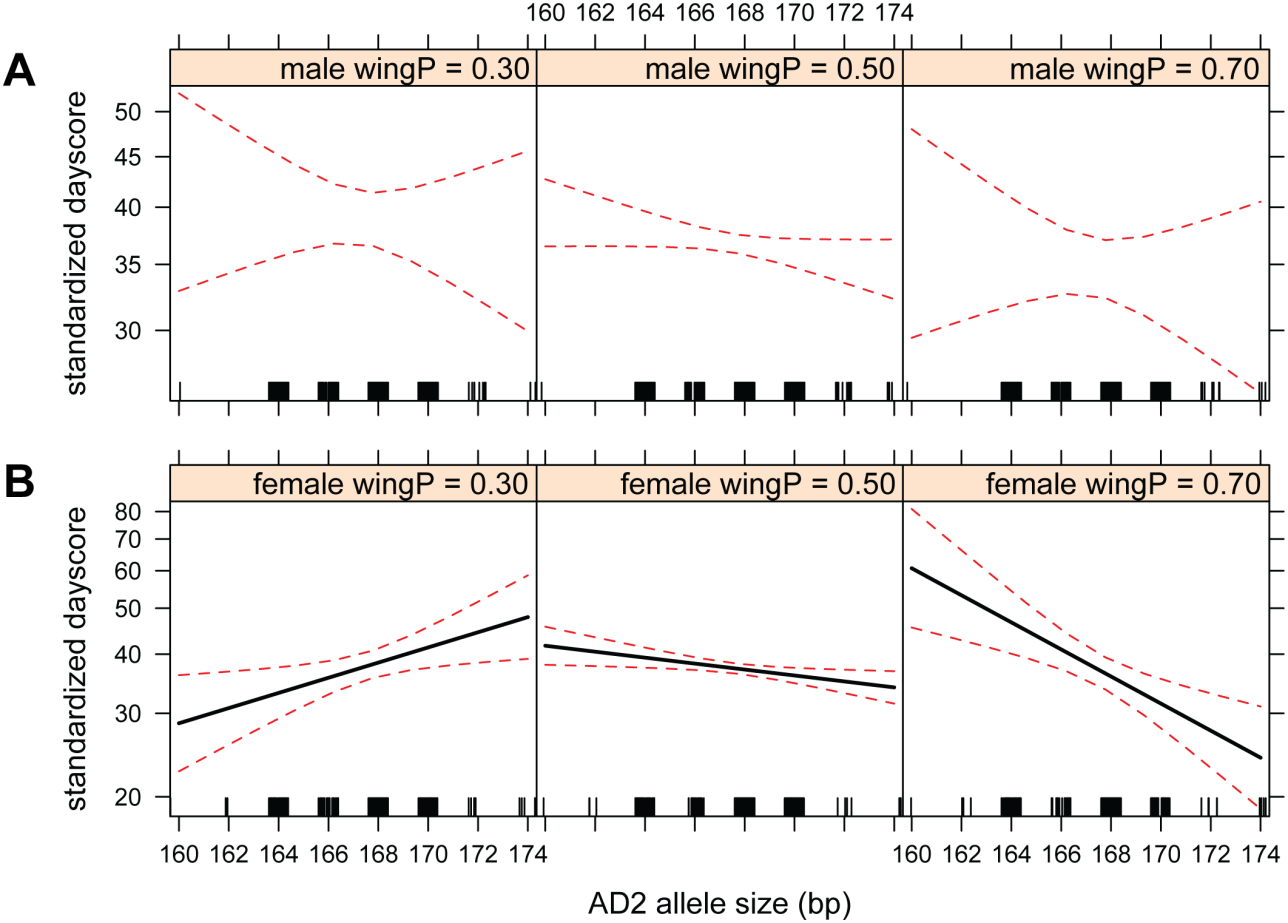
Sex-effect of AD2 allele size x wing pointedness among all populations. Effect of *ADCYAP1* allele size (x-axis) x wing pointedness (Wing P measured as Holynski Index: left = 0.30, center = 0.50, right 0.70) on standardize dayscore (y-axis); lower Holynski indices correspond with more rounded wings (left panels) and greater indices correspond to more pointed wings (right panels). A) males: *p* = 0.552 after FDR (*N* = 389) and B) females: *p* = 0.028 after FDR (*N* = 306). Set 5 Shown.

Two robust genetic patterns were revealed in GLMs and found relatively stable across all five sets (see Methods). Results for Sets 1–4 are found in S3–S6 Tables, while results for Set 5 are presented here (Table 1) as a best representation across sets after considering the false discovery rate: (i) A negative effect on spring arrival (dayscore) was found for the interaction between wing shape and AD2 allele size in females (GLM Set 5: *est* = -0.235 ± 0.076, *p* = 0.028 with FDR; Table 1); females with round wings and short AD2 alleles arrived earlier in the spring, as well as females with pointed wings and long alleles (Fig 2B). (ii) A negative interaction between wing shape and heterozygosity was significant only for females (GLM Set 5: *est* = -1.274 ± 0.384, *p* = 0.028 after FDR; Table 1) as heterozygotes with pointed wing tips arrived significantly earlier than homozygotes with pointed wings and heterozygotes with round wings (Kruskal-Wallis rank sum tests *p* = 0.011 and *p* = 0.005, respectively; S2 Fig). These two main results were generally consistent when a subset of six morphometrically standardized populations were analyzed separately (S7 Table), indicating minimal inter-ringer variation in wing morphology measurements for the full dataset. Although a negative trend between AD2 size and spring arrival was indicated for all blackcaps (i.e. males and females combined) in Set 5 (*est* = -0.009 ± 0.004, *p* = 0.084 after FDR), this pattern was not robust across datasets.

**Table 1:** Generalized linear model fit estimates for all-population analyses (left: all 9 populations, GLM Set 5) and Freiburg intra-population analyses (right: Freiburg, DE).

	All-population Analyses: All 9 Populations Set 5									Intra-population Analyses: Freiburg, DE								
	ALL			MALE			FEMALE			ALL			MALE			FEMALE		
	Est	t value	*P*	Est	t value	*P*	Est	t value	*P*	Est	t value	*P*	Est	t value	*P*	Est	t value	*P*
	± SE		*FDR P*	± SE		*FDR P*	± SE		*FDR P*	± SE		*FDR P*	± SE		*FDR P*	± SE		*FDR P*
**Wing L**	-0.947	-3.12	0.002	-0.673	-1.644	0.100	-1.323	-2.914	0.004	-2.397	-2.408	0.016	-2.019	-1.397	0.162	-2.869	-2.128	0.033
	± 0.304		0.028*	± 0.410		0.300	± 0.454		0.042*	± 0.996		0.329	± 1.445		0.552	± 1.350		0.329
**Wing P**	-0.236	-2.015	0.044	-0.200	-1.283	0.199	-0.299	-1.677	0.094	-0.281	-0.795	0.427	-0.343	-0.646	0.518	-0.157	-0.342	0.733
	± 0.117		0.242	± 0.156		0.522	± 0.178		0.300	± 0.354		0.815	± 0.531		0.851	± 0.460		0.938
**AD1**	0.001	0.146	0.884	-0.005	-0.882	0.378	0.006	1.135	0.256	-0.004	-0.372	0.710	-0.011	-0.685	0.494	0.005	0.366	0.714
	± 0.004		0.928	± 0.005		0.603	± 0.005		0.552	± 0.011		0.938	± 0.016		0.851	± 0.015		0.938
**AD2**	-0.009	-2.574	0.010	-0.010	-1.996	0.046	-0.009	-1.663	0.096	-0.019	-1.637	0.102	-0.034	-2.039	0.042	0.000	-0.028	0.977
	± 0.004		0.084*	± 0.005		0.242	± 0.005		0.300	± 0.011		0.476	± 0.016		0.329	± 0.016		0.992
**meanAD**	-0.006	-1.429	0.153	-0.011	-1.806	0.071	-0.001	-0.216	0.829	-0.015	-1.136	0.256	-0.030	-1.582	0.114	0.004	0.244	0.807
	± 0.004		0.428	± 0.006		0.300	± 0.006		0.928	± 0.013		0.597	± 0.019		0.479	± 0.018		0.968
**het**	-0.021	-1.225	0.221	-0.006	-0.249	0.804	-0.040	-1.653	0.098	-0.087	-1.679	0.093	-0.168	-2.275	0.023	-0.006	-0.087	0.930
	± 0.017		0.546	± 0.024		0.928	± 0.024		0.300	± 0.052		0.476	± 0.074		0.329	± 0.072		0.992
**Wing L X AD1**	-0.132	-0.921	0.357	0.099	0.490	0.624	-0.356	-1.720	0.086	-0.174	-0.354	0.724	-0.696	-1.018	0.309	0.530	0.743	0.457
	± 0.143		0.600	± 0.201		0.873	± 0.207		0.300	± 0.492		0.938	± 0.683		0.683	± 0.712		0.835
**Wing L X AD2**	-0.002	-0.014	0.989	0.038	0.198	0.843	-0.045	-0.209	0.834	0.140	0.307	0.759	-0.876	-1.367	0.171	1.251	1.985	0.047
	± 0.014		0.989	± 0.190		0.928	± 0.214		0.928	± 0.456		0.938	± 0.641		0.552	± 0.631		0.329
**Wing L X meanAD**	-0.089	-0.521	0.602	0.103	0.431	0.667	-0.264	-1.089	0.276	-0.006	-0.011	0.992	-0.971	-1.280	0.201	1.399	1.750	0.080
	± 0.170		0.872	± 0.239		0.875	± 0.243		0.552	± 0.553		0.992	± 0.759		0.563	± 0.800		0.476
**Wing L X het**	0.033	0.052	0.958	0.137	0.160	0.873	-0.182	-0.193	0.847	0.305	0.150	0.881	-1.811	-0.632	0.527	3.395	1.191	0.234
	± 0.633		0.981	± 0.856		0.928	± 0.941		0.928	± 2.027		0.992	± 2.865		0.851	± 2.849		0.578
**Wing P X AD1**	-0.026	-0.461	0.644	0.029	0.360	0.719	-0.070	-0.837	0.402	0.057	0.356	0.722	0.203	0.799	0.425	-0.068	-0.331	0.740
	± 0.057		0.873	± 0.080		0.915	± 0.084		0.603	± 0.160		0.938	± 0.254		0.815	±0.205		0.938
**Wing P X AD2**	-0.057	-1.056	0.291	0.085	1.116	0.265	-0.235	-3.094	0.002	-0.033	-0.202	0.840	0.329	1.384	0.166	-0.459	-2.080	0.038
	± 0.054		0.556	± 0.076		0.552	± 0.076		0.028*	± 0.162		0.980	± 0.237		0.552	± 0.220		0.329
**Wing P X meanAD**	-0.067	-0.996	0.319	0.085	0.859	0.390	-0.206	-2.264	0.024	0.011	0.057	0.954	0.375	1.238	0.216	-0.326	-1.308	0.191
	± 0.067		0.583	± 0.099		0.603	± 0.091		0.168	± 0.194		0.992	± 0.303		0.567	± 0.250		0.563
**Wing P X het**	-0.278	-1.100	0.272	0.308	0.921	0.357	-1.274	-3.318	<0.001	-0.403	-0.557	0.577	0.126	0.119	0.905	-0.943	-0.954	0.340
	± 0.253		0.552	± 0.334		0.600	± 0.384		0.028*	± 0.724		0.898	± 1.055		0.992	± 0.988		0.714

Effects on the response of standardized dayscore are categorized as: Effect of wing shape: measures wing length (log(Wing L)) and wing pointedness (wing P); Effect of genetic variation measures: size of shorter *ADCYAP1* allele (AD1), size of longer *ADCYAP1* allele (AD2), mean *ADCYAP1* allele size (mean AD), and heterozygosity (het); Effect of interaction of wing length and genetic measures; Effect of wing pointedness and genetic measures. Estimate ± Standard Error, *t* value, original *p*-value, and adjusted *p*-value after controlling for FDR listed for each model fit.

* Significant at *p* ≤ 0.10 (FDR)

Mean wing length increased with sampling latitude and longitude among the nine populations considered (nested GLM *est* = 0.002 ± 0.0004, *p* = 0.003). Generalized Linear Model fits revealed a negative relationship between wing length and standardized dayscore (Set 5: *est* = -0.947 ± 0.304, *p* = 0.028 after FDR; Table 1) indicating blackcaps that arrived earlier had longer wings; this effect was present in females (*p* = 0.042), but not in males (*p* = 0.300; Table 1) and consistent with standardized wing morphology results (S7 Table). No relationships were found between mean wing pointedness and sampling latitude or longitude (nested GLM *est* = 0.00002, *p* > 0.05). Eastern migrants had significantly longer and more pointed wings than western migrants (Wilcoxon rank sum tests: *p* = 8.57e-08 and *p* = 0.019, respectively on Set 5; see Fig 1. and [27] for categorization of orientations). In the total dataset, male wing length (mean = 75.56 ± 0.090 mm) was significantly longer than female wing length (mean: 75.27 ± 0.089 mm; Kruskal-Wallis rank sum test *p* = 0.009; S1 Table), which could be a by-product of approximately twice as many males collected in north and east populations compared to females (Os, Bw, Ry; see S1 Table); wing pointedness did not differ between sexes (*p* > 0.05). Arrival day was similar for males (mean: 36.71 ± 0.383) and females (mean: 37.55 ± 0.422) among all nine populations (Kruskal-Wallis rank sum test *p* = 0.251), as well as within each individual population (*p* > 0.05). A year effect on dayscore was apparent for both males (*p* = 0.043) and females (*p* = 0.002), presumably influenced by variation in passage dates among years (S1 Table).

### Effects within the Freiburg population

Individual predictor variables (wing L, wing P, AD1, AD2, and meanAD) did not differ between sexes within the Freiburg, Germany population (2007–2011), with the exception that males were less heterozygous at *ADCYAP1* (mean heterozygosity = 0.633 ± 0.034) than females (mean heterozygosity = 0.754 ± 0.031; Wilcox rank sum test *p* = 0.009; S1 Table). Compared to the all-population results, none of the effects of wing morphology and *ADCYAP1* variation on arrival date were robust within the Freiburg population after controlling for false discovery rate (all *p* > 0.10; Table 1).

**Fig 3:**
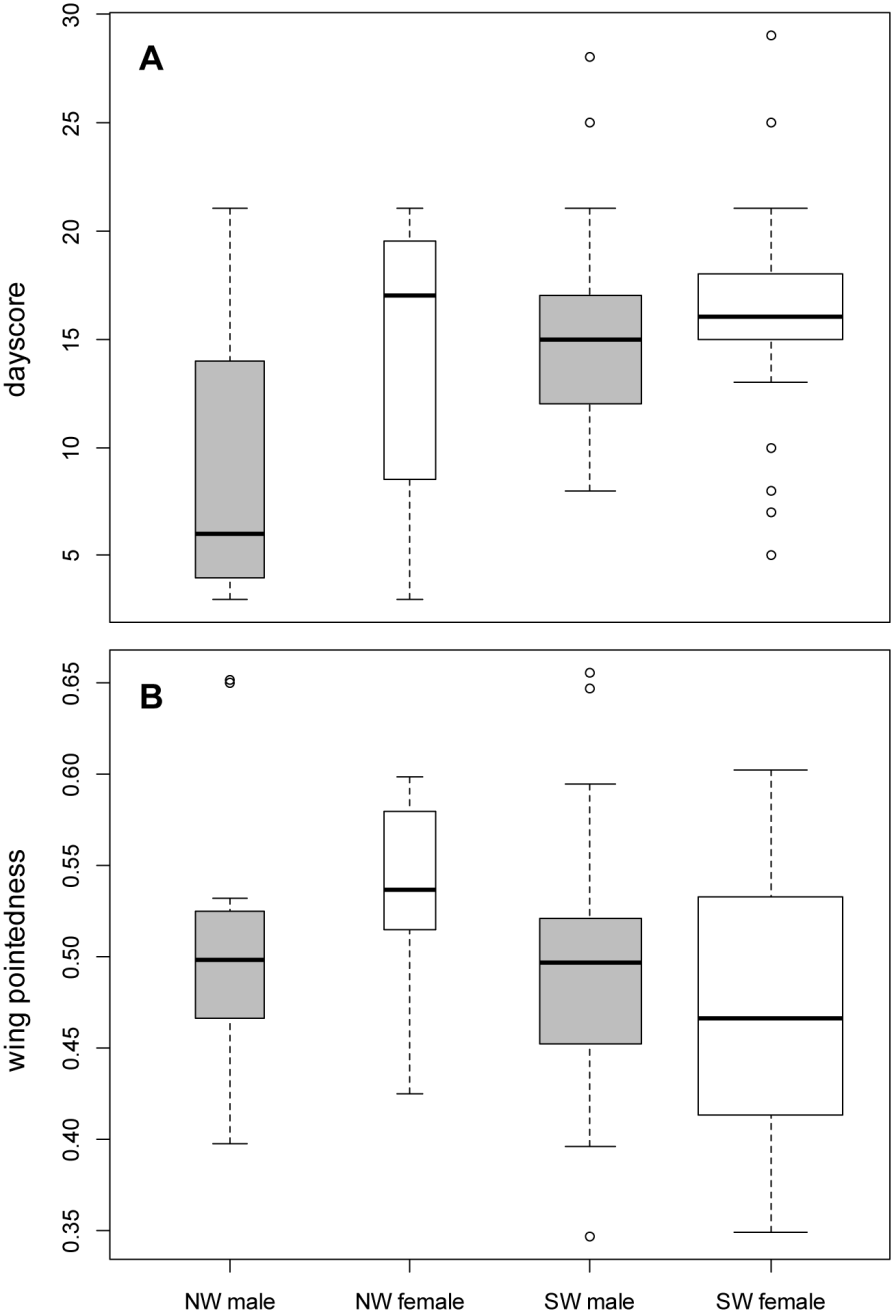
Freiburg 2011 comparison of migratory orientations based on sex and stable isotope (*δ* ^*2*^ *H*) assignment to wintering grounds. NW males *(N* = 13), NW females (*N* = 7), SW males (*N* = 28), SW females (*N* = 50). Width of box is proportional to sample size. A) Dayscore: NW males arrived significantly earlier than SW males (*p* = 0.009) and SW females (*p* = 0.004). B) Wing pointedness (Holynski Index): NW females had significantly more pointed wings than SW females (*p* = 0.026). A detailed description of stable isotope analysis and geographic assignments is found in [27].

The combined data from Freiburg 2007–2011 reveal a bimodality in male arrival dates (by separating sexes, S3 Fig) as well as bimodal *ADCYAP1* allele size frequencies in this population (sexes combined, S1 Fig). A positive relationship between individual stable isotopes *δ* ^*2*^ *H* values and dayscore in spring 2011 indicated that NW-migrants arrived first to the Freiburg breeding grounds (Spearman Rank Test: *rho* = 0.309, *p* = 0.0026). NW-migrating males arrived significantly earlier than both SW-migrating males (Wilcoxon Rank Sum Test: *p* = 0.009) and SW females (*p* = 0.004; Fig 3A). No differences were found among the groups for wing length (*p* > 0.05), while the wings of NW females were more pointed compared to those of SW females (*p* = 0.026, Fig 3B). No differences were found in *ADCYAP1* allele size among these groups.

Stable isotopes (*δ* ^*2*^ *H*) were positively correlated among males and females paired with each other (*N* = 23 total pairs) during 2010 and 2011 (*r* ^*2*^ = 0.588, *p* = 0.003), although this test statistic falls within the confidence interval (0.263–0.729) produced from the null model of 1000 random pairs; thus the relationship between male and female *δ* ^*2*^ *H* was not different from random. Wing morphology and *ADCYAP1* allele size predictors were not found to correlate between males and females in breeding pairs, with the exception of a negative relationship between AD1 allele size between sexes (*r* ^*2*^ = -0.470, *p* = 0.027): females with short AD1 alleles paired with long-allele males and vice versa; this test statistic was found to lie outside the confidence interval (-0.423–0.426) produced by 1000 randomized pairs in the null model and is thus different than random and indicative of negative assortment by AD1 allele size.

## Discussion

### Effects of wing morphology on spring arrival in blackcaps

Wing length and pointedness served as proxies for migratory distance in our blackcap study, as increases in each correspond to greater migratory distance [16]. Across the nine blackcap populations sampled, mean wing length increased with sampling latitude and longitude. Blackcaps following westerly routes to the wintering grounds (i.e. west of the W/E migratory divide at approximately 13°E, see [27]) had earlier spring arrival dates compared to individuals wintering further east. Longer wing lengths were found to predict earlier spring arrival for blackcaps across nine migratory populations in Europe, a pattern which appears to be driven by females. Without additionally constraining wing measurements for allometry [15,48], wing length may correspond better to the isometric size (and weight) differences among birds (i.e. larger birds arrive earlier). However, wing shape (measured here as Holynski Index) may better reflect migratory behavior rather than size differences [15,16]. Yet single-variable wing shape analyses did not provide us conclusive results across the populations considered; these results contradict our initial expectation, which predicted early arriving blackcaps would have wing morphologies typical of birds traveling shorter distances (i.e. shorter, rounder wings as in [10,17]). Considering the pattern specific to blackcaps [16] and general among passerines [18,49,50], that longer and more pointed wings are associated with longer migratory distances, we conclude that early-arriving blackcaps with longer wings are most likely longer-distance migrants in our study.

### Female-specific interaction effects of ADCYAP1 and wing morphology

The *ADCYAP1* locus is suggested to be associated with migratory activity via adaptive allele frequency shifts [22], linking behaviors associated with seasonal movements to a functional locus. Our results support this prevailing hypothesis and add new insights into how genetic variation at this locus and wing morphology may interact to affect spring migration phenology among nine wild blackcap populations in Europe. Spring arrival of female blackcaps correlated with the interaction between wing pointedness and *ADCYAP1* allele length (of the longer allele, AD2) and differed depending on wing phenotype. For females with pointed wings, longer alleles predicted early arrival, whereas in round-winged females shorter alleles predict earlier arrival. Furthermore, females heterozygous at *ADCYAP1* with pointed wing tips arrived significantly earlier than both homozygotes with pointed wings and heterozygotes with round wings. Therefore the association between wing shape and *ADCYAP1* genotype appears to be female-specific across the range of this species and predicts spring arrival date when predictor variables are considered together (yet not alone). Similarly, a female-specific interaction between *ADCYAP1* genotype and breeding latitude was shown to affect laying date in a wild tree swallow (*Tachycineta bicolor*) population [26], therefore genotype-environment interactions (GxE) may be important to consider when studying candidate genes for migration.

The observed interplay between migratory phenotype and *ADCYAP1* genotype on blackcap spring arrival may reflect trait divergence among specific migratory groups. Morphotypes associated with different migratory orientation may discriminate among breeding habitats allowing morphological adaptations for flight (e.g. mobility and maneuverability) to evolve rapidly in response to habitat change [51]. If divergent selection for habitat further promotes the evolution of genetically-based differences between populations [52], we then expect these effects to be apparent in both sexes. Yet we find *ADCYAP1* x wing shape interaction effects only in female blackcaps in this study, contradictory to our expectation (stronger male effects). Alternatively, sex-specific morphotypes may be antagonistically selected via intralocus sexual conflict, which may constrain evolution toward sex-specific optima for traits such as wing shape and length if these traits important for migration are controlled by genes with pleiotropic effects [53]. Sex roles associated with defense of the breeding territory (e.g. early arrival) have been well established as a migratory adaptation in males and protandrous blackcaps, while female condition and timing may be optimized for reproduction and adapted to the local breeding environment or stop over sites en route and thus partially explain our sex-specific results here [1,54,55]. Northern wheatears (*Oenanthe oenanthe leucorhoa*) have been shown to display differences in the timing of spring migration between males and females, likely due to different migration strategies: males are risk-takers and adjust fuel deposition rates en route or completely by-pass stop over sites to optimize migration time (time-minimization strategy), while females display a strategy more dependent on refueling at stop over sites to ensure sufficient energy storage for reproduction after arrival to the breeding ground [55].

### Spring arrival in Freiburg, DE

Within the large sample size of the Freiburg population, interactions between *ADCYAP1* allele size and wing length did not affect spring arrival. Possibly, interactions among *ADCYAP1* allele size and wing length are masked by the fact that two genetically diverging populations mix here [10,27]. Alternatively, the selective pressures on spring arrival may vary here from other populations given that spring arrival is likely to contribute to assortative mating and thus to the genetic divergence of the two populations [10,45]. At the Freiburg breeding ground, NW-migrants (travelling from the British Isles) tend to arrive earlier in the spring compared to SW-migrants (travelling from SW Mediterranean), confirmed both in our 2011 dataset and previously from 2006–2007 [10].

Interestingly, we found no evidence of positive assortative mating based upon *ADCYAP1* allele matching among males and females in breeding pairs in this study. In fact, allele mis-match (i.e. negative assortment) among pairs was significant for AD1 allele size, which was not associated with spring arrival in our study. Compared to previous findings of Bearhop et al. [56], we found no evidence of positive assortment based on stable isotopes, *ADCYAP1* genotypes, or wing morphology of male and female blackcaps in pairs (Freiburg, DE). Alternatively, microhabitat choice among blackcaps has been proposed to be associated with migratory orientation [45]. For example, NW-migrants tended to prefer microhabitats with open understories while SW-migrants prefer habitats with dense understories [57]; in line with observations by Hermes et al. [57], we found that NW-migrant females had more pointed wings compared to rounder wing shapes of female SW-migrants (Freiburg 2011).

By integrating stable isotopes from a sample of blackcaps in Freiburg captured in 2011, our results again contradict our expectation (i.e. NW-migrants should have rounder wings than SW migrants, based on patterns described by [17]) and instead indicate that NW-migrants had more pointed wings compared to SW-migrants, especially being true for females. If wing shape is indeed indicative of migratory distance, our results here suggest NW-migrants traveled longer distances than SW-migrants in 2011, which may reflect an expansion of blackcaps’ wintering range, utilization of indirect migration routes [58], or simply be an effect visible in one year (2011) but not the previous ones [17]. Alternatively, blackcaps following the traditional SW route may be evolving shorter migratory distances (e.g. overwintering in France instead of Spain) and shorter/rounder wings due to a reduction in migratory behavior, a hypothesis consistent with that of Pulido and Berthold [7]. In support, several presumably SW-migrant blackcaps (with orientations from Freiburg ranging from 200–220°) have recently attempted to overwinter in France (47–299 km away) or within the immediate region of Freiburg (18 km away, from ring recovery data).

The evolution of behavioral traits involved in migration (i.e. timing, direction, distance) may in turn cause shifts in other correlated traits inherited with the ‘migratory gene package’ [3,5,59], e.g. life history, physiological, or morphological traits. Such traits may be either adaptations or exaptations for migration [1]. Although *ADCYAP1* was found to lie outside prominent islands of genomic divergence or speciation when examined across the Swainson’s thrush (*Catharus ustulatus*) migratory divide/hybrid zone [14,60], this candidate gene for migration still appears to be but one of many ‘multifaceted’ mechanisms involved in the evolutionary processes (e.g. selective sweeps) promoting genetic divergence [14,60]. While controversy remains on the hypothesized existence of an avian migratory syndrome or a syndrome common across animal taxa [1,3,59], we here provide a better understanding of the underlying genetic interactions associated with blackcap migration from wild populations. The link between the *ADCYAP1* candidate gene and morphology related to migration is likely indirect, yet effects may be inter-related with migratory behaviors (e.g. timing, direction, distance) within a common migratory suite via epistatic interactions and observed to covary given a precise selective regime.

## Conclusion

Among wild blackcaps, we found a relationship between the interaction of wing pointedness and *ADCYAP1* allele size in females: females with rounder wings arrived earlier if they had shorter *ADCYAP1* alleles, while females with more pointed wings and longer alleles arrived earlier.

An association between wing shape and *ADCYAP1* allele size therefore indicates that genetic variation exists between different morphotypes (e.g. round vs. pointed wings) and affects spring arrival time in wild blackcaps. The combined effects of genotype and phenotype on spring arrival (i.e. wing shape x AD2 allele size) may promote allochrony between blackcaps with different migratory behaviors if these traits are linked and inherited together in a common migratory package, which is still poorly understood. Incorporating the dynamics of migratory orientation and wing shape in a well-studied population of blackcaps in Freiburg, Germany helps explain the observed effect on spring arrival: while males face selection pressure during migration for early arrival (NW-migrants precede SW-migrants), the interaction of *ADCYAP1* genotypes and wing shape phenotypes adapted to particular migratory behaviors and/or habitats may better predict female spring arrival to the breeding grounds, and thus reproductive phenology and success.

## Supporting Information

S1 Fig *ADCYAP1* allele frequency histograms (allele size in base pairs) per population. Population codes: **Ub** = Uebersyren, LU; **Fr** = Freiburg, DE; **Rz** = Radolfzell, DE; **Os** = Oslo, NO; **Kf** = Kefermarkt, AT; **Vn** = Vienna, AT; **Ry** = Rybachy, RU; **Bw** = Białowieża Forest, PL; **Km** = Kalimok, BG. Geographic coordinates for collecting localities can be found in S1 Table.
(TIF)

S2 Fig Female effect of wing shape (pointed vs. round) x heterozygosity (homozygote vs. heterozygote) on spring arrival (dayscore). P = pointed wing tips (blue, Wing P > 0.5346), 1st quartile of Holynski Index distribution; R = round wing tips (green, Wing P < 0.4431), 4th quartile of Holynksi Index distribution; Hom = homozygote and Het = heterozygote at *ADCYAP1* locus. P-Het (*N* = 44) arrived significantly earlier than P-Hom (*N* = 22, *p* = 0.011 Kruskal Wallis Rank Sum Test) and also arrived earlier than R-Het (*N* = 49, *p* = 0.005). No difference in arrival date between R-Hom (*N* = 18) and R-Het, or remaining comparisons (*p* > 0.05).
(TIF)

S3 Fig Density plots of Freiburg dayscores for males (A) and females (B) from 2007–2011. (TIF)

S1 Table Mean statistics for blackcap populations. A) all blackcaps; B) male blackcaps; C) female blackcaps. Population codes coincide with Fig 1 and S1 Fig. Geographic location of each sampling population in decimal degrees (lat °E/long °N); Total sample size (*N* total); mean wing length (wing L); mean wing pointedness determined with Holynski Index (wing P); mean size of shorter *ADCYAP1* allele (AD1); mean size of longer *ADCYAP1* allele (AD2); mean size of both *ADCYAP1* alleles (meanAD); mean heterozygosity (het); mean capture date per population with 1 = March 19 (raw date).
(DOC)

S2 Table *ADCYAP1* Allele frequencies by population (sample size) and mean allele frequencies across all populations. (DOC)

S3 Table Generalized linear model fit estimates for all 9 populations, Set 1. Effects on standardized dayscore are categorized as: Effect of wing shape: measures wing length (log(Wing L)) and wing pointedness (wing P); Effect of genetic variation measures: size of shorter *ADCYAP1* allele (AD1), size of longer *ADCYAP1* allele (AD2), mean *ADCYAP1* allele size (mean AD), and heterozygosity (het); Effect of interaction of wing length and genetic measures; Effect of wing pointedness and genetic measures. Estimate ± Standard Error, *t* value, *p*-value, and *p*-value after FDR listed for each model fit.
(DOC)

S4 Table Generalized linear model fit estimates for all 9 populations, Set 2. See S3 Table for details.
(DOC)

S5 Table Generalized linear model fit estimates for all 9 populations, Set 3. See S3 Table for details.
(DOC)

S6 Table Generalized linear model fit estimates for all 9 populations, Set 4. See S3 Table for details.
(DOC)

S7 Table Generalized linear model fit estimates for 6 morphometrically standardized populations. See S3 Table for details.
(DOC)
