# Supplementary figures and images for: Interactions between a Candidate Gene for Migration (ADCYAP1), Morphology and Sex Predict Spring Arrival in Blackcap Populations
Source: PLoS One. 2015 Dec 18;10(12):e0144587. doi: 10.1371/journal.pone.0144587 (PMC4684316; doi:10.1371/journal.pone.0144587)

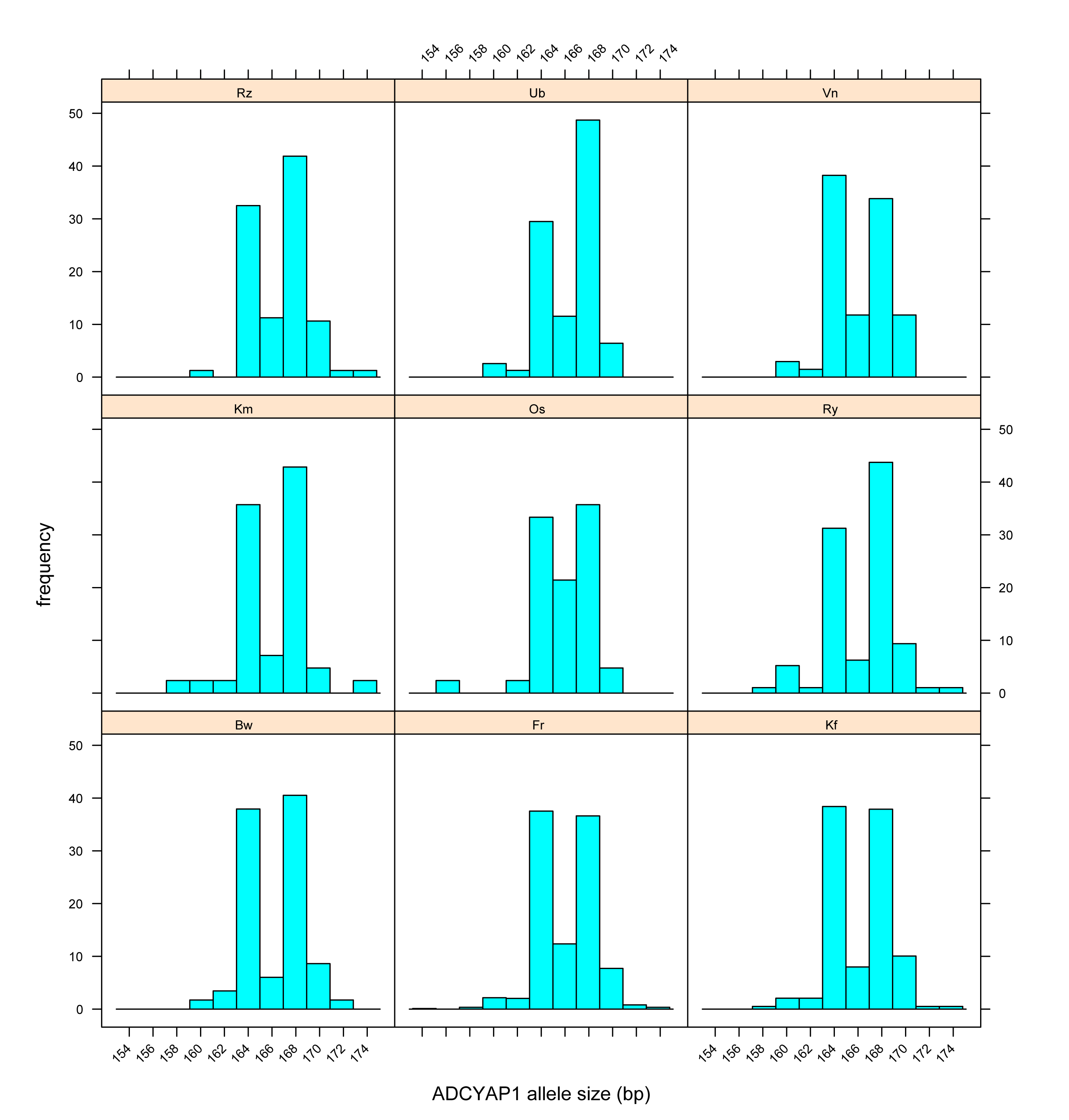

Supplement: S1 Fig — Population codes: Ub = Uebersyren, LU; Fr = Freiburg, DE; Rz = Radolfzell, DE; Os = Oslo, NO; Kf = Kefermarkt, AT; Vn = Vienna, AT; Ry = Rybachy, RU; Bw = Białowieża Forest, PL; Km = Kalimok, BG. Geographic coordinates for collecting localities can be found in S1 Table. (TIF) [file pone.0144587.s001.tif]

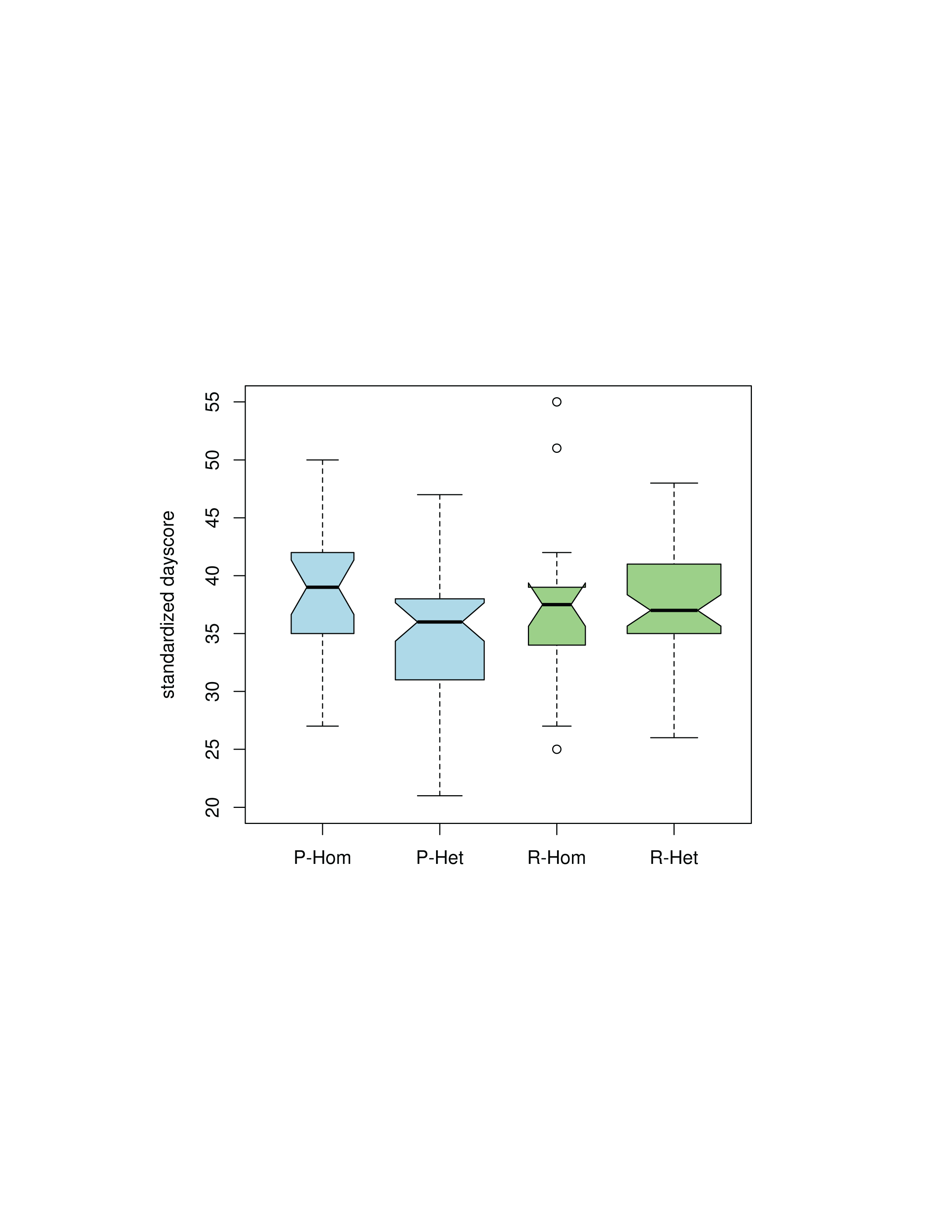

Supplement: S2 Fig — P = pointed wing tips (blue, Wing P > 0.5346), 1st quartile of Holynski Index distribution; R = round wing tips (green, Wing P < 0.4431), 4th quartile of Holynksi Index distribution; Hom = homozygote and Het = heterozygote at ADCYAP1 locus. P-Het (N = 44) arrived significantly earlier than P-Hom (N = 22, p = 0.011 Kruskal Wallis Rank Sum Test) and also arrived earlier than R-Het (N = 49, p = 0.005). No difference in arrival date between R-Hom (N = 18) and R-Het, or remaining comparisons (p > 0.05). (TIF) [file pone.0144587.s002.tif]

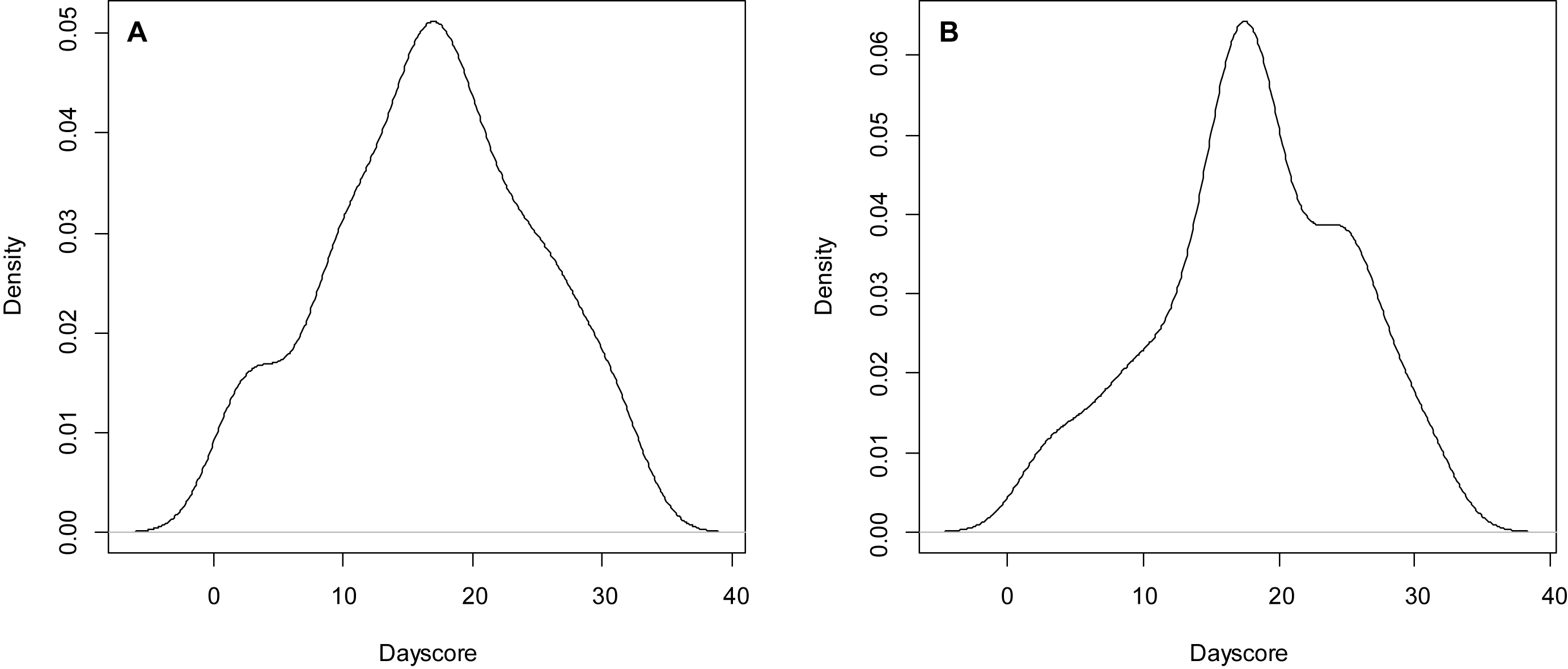

Supplement: S3 Fig — (TIF) [file pone.0144587.s003.tif]
