# Supplementary material for: Interactions between a Candidate Gene for Migration (ADCYAP1), Morphology and Sex Predict Spring Arrival in Blackcap Populations
Source: PLoS One. 2015 Dec 18;10(12):e0144587. doi: 10.1371/journal.pone.0144587 (PMC4684316; doi:10.1371/journal.pone.0144587)
Supplement: S1 Table — A) all blackcaps; B) male blackcaps; C) female blackcaps. Population codes coincide with Fig 1 and S1 Fig. Geographic location of each sampling population in decimal degrees (lat °E/long °N); Total sample size (N total); mean wing length (wing L); mean wing pointedness determined with Holynski Index (wing P); mean size of shorter ADCYAP1 allele (AD1); mean size of longer ADCYAP1 allele (AD2); mean size of both ADCYAP1 alleles (meanAD); mean heterozygosity (het); mean capture date per population with 1 = March 19 (raw date). (DOC) [file pone.0144587.s004.doc]

**S1 Table.**

|  | **code** | **population** | ***N* total** | **year(s) sampled** | **lat** | **long** | **wing L** | **wing P** | **AD1** | **AD2** | **meanAD** | **het** | **raw dayscore** |
| --- | --- | --- | --- | --- | --- | --- | --- | --- | --- | --- | --- | --- | --- |
| A. | Ub | Uebersyren, LU | 39 | 2011 | 49.63 | 6.28 | 75.26 | 0.51 | 165.18 | 167.69 | 166.44 | 0.67 | 12 |
|  | Fr | Freiburg, DE | 441 | 2007-2011 | 48.03 | 7.82 | 75.00 | 0.48 | 164.80 | 167.40 | 166.10 | 0.68 | 22 |
|  | Rz | Radolfzell, DE | 80 | 2006 | 47.75 | 8.98 | 75.32 | 0.48 | 165.48 | 167.95 | 166.71 | 0.68 | 25 |
|  | Os | Oslo, NO | 21 | 2003, 2006, 2008, 2010, 2011 | 59.55 | 11.27 | 76.67 | NA | 164.30 | 167.50 | 165.90 | 0.81 | 53 |
|  | Kf | Kefermarkt, AT | 194 | 2010-2011 | 48.44 | 14.54 | 75.37 | 0.48 | 164.80 | 167.60 | 166.20 | 0.70 | 39 |
|  | Vn | Vienna, AT | 34 | 2010 | 48.22 | 16.28 | 75.96 | 0.51 | 164.60 | 167.70 | 166.10 | 0.74 | 61 |
|  | Ry | Rybachy, RU | 48 | 2011 | 55.15 | 20.85 | 76.86 | 0.51 | 164.80 | 167.90 | 166.30 | 0.79 | 35 |
|  | Bw | Białoweiża, PL | 58 | 2011 | 52.70 | 23.87 | 76.86 | 0.53 | 164.80 | 167.70 | 166.30 | 0.72 | 41 |
|  | Km | Kalimok, BG | 21 | 2010-2011 | 44.00 | 26.26 | 77.29 | NA | 164.70 | 167.50 | 166.10 | 0.71 | 50 |
| B. | Ub | Uebersyren, LU | 27 | 2011 | 49.63 | 6.28 | 75.43 | 0.51 | 164.70 | 167.40 | 166.10 | 0.67 | 12 |
|  | Fr | Freiburg, DE | 229 | 2007-2011 | 48.03 | 7.82 | 75.05 | 0.48 | 164.90 | 167.30 | 166.10 | 0.64 | 22 |
|  | Rz | Radolfzell, DE | 33 | 2006 | 47.75 | 8.98 | 75.02 | 0.47 | 165.00 | 168.10 | 166.50 | 0.76 | 23 |
|  | Os | Oslo, NO | 20 | 2003, 2006, 2008, 2010, 2011 | 59.55 | 11.27 | 77.00 | NA | 164.30 | 167.50 | 165.90 | 0.80 | 52 |
|  | Kf | Kefermarkt, AT | 103 | 2010-2011 | 48.44 | 14.54 | 75.41 | 0.48 | 164.70 | 167.30 | 166.00 | 0.68 | 39 |
|  | Vn | Vienna, AT | 26 | 2010 | 48.22 | 16.28 | 76.21 | 0.52 | 164.50 | 167.60 | 166.10 | 0.65 | 61 |
|  | Ry | Rybachy, RU | 33 | 2011 | 55.16 | 20.84 | 77.12 | 0.52 | 164.70 | 168.20 | 166.50 | 0.82 | 35 |
|  | Bw | Białoweiża, PL | 38 | 2011 | 52.70 | 23.87 | 77.16 | 0.53 | 164.70 | 167.70 | 166.20 | 0.74 | 42 |
|  | Km | Kalimok, BG | 9 | 2010-2011 | 44.05 | 26.52 | 77.56 | NA | 164.90 | 168.00 | 166.40 | 0.78 | 46 |
| C. | Ub | Uebersyren, LU | 12 | 2011 | 49.63 | 6.28 | 74.88 | 0.50 | 166.20 | 168.30 | 167.20 | 0.67 | 12 |
|  | Fr | Freiburg, DE | 212 | 2007-2011 | 48.03 | 7.82 | 74.94 | 0.47 | 164.80 | 167.40 | 166.10 | 0.73 | 23 |
|  | Rz | Radolfzell, DE | 47 | 2006 | 47.75 | 8.98 | 75.53 | 0.49 | 165.80 | 167.80 | 166.80 | 0.62 | 25 |
|  | Os* | Oslo, NO | 1 | 2008 | 59.55 | 11.27 | 76 | NA | 164.00 | 168.00 | 166.00 | 1.00 | 69 |
|  | Kf | Kefermarkt, AT | 91 | 2010-2011 | 48.44 | 14.54 | 75.3 | 0.48 | 165.00 | 167.90 | 166.50 | 0.73 | 39 |
|  | Vn | Vienna, AT | 8 | 2010 | 48.22 | 16.28 | 75.12 | 0.47 | 164.80 | 168.00 | 166.40 | 1.00 | 60 |
|  | Ry | Rybachy, RU | 15 | 2011 | 55.16 | 20.84 | 76.3 | 0.48 | 164.80 | 167.30 | 166.10 | 0.73 | 35 |
|  | Bw | Białoweiża, PL | 20 | 2011 | 52.7 | 23.87 | 76.3 | 0.54 | 164.90 | 167.80 | 166.40 | 0.70 | 39 |
|  | Km | Kalimok, BG | 12 | 2011 | 44.05 | 26.52 | 77.08 | NA | 164.50 | 167.20 | 165.80 | 0.67 | 52 |

* The Os population was not included in analyses on female population means due to only one individual represented

**S2 Table.**
