# Supplementary material for: Interactions between a Candidate Gene for Migration (ADCYAP1), Morphology and Sex Predict Spring Arrival in Blackcap Populations
Source: PLoS One. 2015 Dec 18;10(12):e0144587. doi: 10.1371/journal.pone.0144587 (PMC4684316; doi:10.1371/journal.pone.0144587)
Supplement: S2 Table — (DOC) [file pone.0144587.s005.doc]

**S2 Table.**

| **Allele (bp)** | **Km (21)** | **Fr (441)** | **Kf (194)** | **Ub (39)** | **Os (21)** | **Bw (58)** | **Rz (80)** | **Ry (48)** | **Vn (34)** | **Mean** |
| --- | --- | --- | --- | --- | --- | --- | --- | --- | --- | --- |
| **154** | 0.000 | 0.001 | 0.000 | 0.000 | 0.000 | 0.000 | 0.000 | 0.000 | 0.000 | 0.000 |
| **156** | 0.000 | 0.000 | 0.000 | 0.000 | 0.024 | 0.000 | 0.000 | 0.000 | 0.000 | 0.003 |
| **158** | 0.024 | 0.004 | 0.005 | 0.000 | 0.000 | 0.000 | 0.000 | 0.010 | 0.000 | 0.005 |
| **160** | 0.024 | 0.021 | 0.021 | 0.026 | 0.000 | 0.017 | 0.013 | 0.052 | 0.029 | 0.023 |
| **162** | 0.024 | 0.023 | 0.021 | 0.013 | 0.024 | 0.034 | 0.000 | 0.010 | 0.015 | 0.018 |
| **164** | 0.357 | 0.378 | 0.384 | 0.295 | 0.333 | 0.379 | 0.325 | 0.313 | 0.382 | 0.349 |
| **166** | 0.071 | 0.117 | 0.080 | 0.115 | 0.214 | 0.060 | 0.113 | 0.063 | 0.118 | 0.106 |
| **168** | 0.429 | 0.367 | 0.379 | 0.487 | 0.357 | 0.405 | 0.419 | 0.438 | 0.338 | 0.402 |
| **170** | 0.048 | 0.077 | 0.101 | 0.064 | 0.048 | 0.086 | 0.106 | 0.094 | 0.118 | 0.082 |
| **172** | 0.000 | 0.009 | 0.005 | 0.000 | 0.000 | 0.017 | 0.013 | 0.010 | 0.000 | 0.006 |
| **174** | 0.024 | 0.004 | 0.005 | 0.000 | 0.000 | 0.000 | 0.013 | 0.010 | 0.000 | 0.006 |
