# Supplementary material for: Interactions between a Candidate Gene for Migration (ADCYAP1), Morphology and Sex Predict Spring Arrival in Blackcap Populations
Source: PLoS One. 2015 Dec 18;10(12):e0144587. doi: 10.1371/journal.pone.0144587 (PMC4684316; doi:10.1371/journal.pone.0144587)
Supplement: S3 Table — Effects on standardized dayscore are categorized as: Effect of wing shape: measures wing length (log(Wing L)) and wing pointedness (wing P); Effect of genetic variation measures: size of shorter ADCYAP1 allele (AD1), size of longer ADCYAP1 allele (AD2), mean ADCYAP1 allele size (mean AD), and heterozygosity (het); Effect of interaction of wing length and genetic measures; Effect of wing pointedness and genetic measures. Estimate ± Standard Error, t value, p-value, and p-value after FDR listed for each model fit. (DOC) [file pone.0144587.s006.doc]

**S3 Table.**

|  | **All-population Analyses: All 9 Populations Set 1** | | | | | | | | |
| --- | --- | --- | --- | --- | --- | --- | --- | --- | --- |
|  | **ALL** | | | **MALE** | | | **FEMALE** | | |
|  | **Est ± SE** | **t value** | ***P, FDR P*** | **Est ± SE** | **t value** | ***P, FDR P*** | **Est ± SE** | **t value** | ***P, FDR P*** |
| **Wing L** | -0.697 ± 0.310 | -2.245 | 0.025, 0.210 | -0.695 ± 0.414 | -1.677 | 0.094, 0.395 | -0.693 ± 0.474 | -1.464 | 0.143, 0.415 |
| **Wing P** | -0.163 ± 0.118 | -1.384 | 0.166, 0.415 | -0.175 ± 0.157 | -1.113 | 0.266, 0.559 | -0.160 ± 0.180 | -0.889 | 0.374, 0.683 |
| **AD1** | -0.001 ± 0.004 | -0.371 | 0.710, 0.806 | -0.009 ± 0.005 | -1.797 | 0.072, 0.373 | 0.007 ± 0.005 | 1.447 | 0.148, 0.415 |
| **AD2** | -0.008 ± 0.004 | -1.992 | 0.046, 0.322 | -0.010 ± 0.005 | -1.889 | 0.059, 0.354 | -0.005 ± 0.006 | -0.818 | 0.413, 0.723 |
| **meanAD** | -0.006 ± 0.004 | -1.379 | 0.168, 0.415 | -0.014 ± 0.006 | -2.280 | 0.023, 0.210 | 0.003 ± 0.006 | 0.446 | 0.656, 0.787 |
| **het** | -0.013 ± 0.017 | -0.781 | 0.435, 0.731 | 0.010 ± 0.024 | 0.412 | 0.680, 0.793 | -0.043 ± 0.025 | -1.75 | 0.080, 0.373 |
| **Wing L X AD1** | 0.040 ± 0.146 | 0.274 | 0.784, 0.823 | 0.202 ± 0.199 | 1.013 | 0.311, 0.594 | -0.114 ± 0.219 | -0.519 | 0.603, 0.762 |
| **Wing L X AD2** | 0.091 ± 0.154 | 0.592 | 0.554, 0.757 | -0.005 ± 0.201 | -0.025 | 0.980, 0.980 | 0.256 ± 0.240 | 1.067 | 0.286, 0.572 |
| **Wing L X meanAD** | 0.090 ± 0.179 | 0.500 | 0.617, 0.762 | 0.148 ± 0.244 | 0.606 | 0.545, 0.757 | 0.055 ± 0.264 | 0.209 | 0.835, 0.855 |
| **Wing L X het** | 0.3639 ± 0.658 | 0.553 | 0.580, 0.761 | -0.266 ± 0.886 | -0.300 | 0.764, 0.823 | 1.127 ± 0.985 | 1.145 | 0.252, 0.559 |
| **Wing P X AD1** | -0.036 ± 0.055 | -0.648 | 0.517, 0.757 | 0.045 ± 0.077 | 0.584 | 0.559, 0.757 | -0.094 ± 0.084 | -1.124 | 0.261, 0.559 |
| **Wing P X AD2** | -0.090 ± 0.056 | -1.604 | 0.109, 0.415 | 0.052 ± 0.078 | 0.665 | 0.506, 0.757 | -0.280 ± 0.081 | -3.450 | < 0.001, 0.042* |
| **Wing P X meanAD** | -0.101 ± 0.067 | -1.507 | 0.132, 0.415 | 0.062 ± 0.096 | 0.641 | 0.522, 0.757 | -0.256 ± 0.094 | -2.709 | 0.007, 0.098* |
| **Wing P X het** | -0.369 ± 0.258 | -1.433 | 0.152, 0.415 | 0.094 ± 0.340 | 0.276 | 0.782, 0.823 | -1.206 ± 0.392 | -3.077 | 0.002, 0.042* |

* Significant at *p* ≤ 0.10 (FDR)
