# Supplementary material for: Interactions between a Candidate Gene for Migration (ADCYAP1), Morphology and Sex Predict Spring Arrival in Blackcap Populations
Source: PLoS One. 2015 Dec 18;10(12):e0144587. doi: 10.1371/journal.pone.0144587 (PMC4684316; doi:10.1371/journal.pone.0144587)
Supplement: S4 Table — See S3 Table for details. (DOC) [file pone.0144587.s007.doc]

**S4 Table.**

|  | **All-population Analyses: All 9 Populations Set 2** | | | | | | | | |
| --- | --- | --- | --- | --- | --- | --- | --- | --- | --- |
|  | **ALL** | | | **MALE** | | | **FEMALE** | | |
|  | **Est ± SE** | **t value** | ***P, FDR P*** | **Est ± SE** | **t value** | ***P, FDR P*** | **Est ± SE** | **t value** | ***P, FDR P*** |
| **Wing L** | -1.065 ± 0.318 | -3.352 | < 0.001, 0.014* | -0.979 ± 0.429 | -2.280 | 0.023, 0.121 | -1.130 ± 0.478 | -2.365 | 0.018, 0.108 |
| **Wing P** | -0.249 ± 0.121 | -2.056 | 0.040 , 0.153 | -0.279 ± 0.162 | -1.665 | 0.096, 0.288 | -0.216 ± 0.182 | -1.186 | 0.236, 0.451 |
| **AD1** | 0.003 ± 0.004 | 0.734 | 0.463, 0.648 | -0.002 ± 0.005 | -0.297 | 0.767, 0.849 | 0.007 ± 0.005 | 1.310 | 0.190, 0.399 |
| **AD2** | -0.006 ± 0.004 | -1.449 | 0.147, 0.373 | -0.007 ± 0.005 | -1.377 | 0.169, 0.394 | -0.004 ± 0.006 | -0.667 | 0.505, 0.684 |
| **meanAD** | -0.002 ± 0.004 | -0.397 | 0.691, 0.806 | -0.007 ± 0.006 | -1.039 | 0.299, 0.502 | 0.003 ± 0.006 | 0.427 | 0.669, 0.803 |
| **het** | -0.017 ± 0.018 | -0.933 | 0.351, 0.534 | -0.005 ± 0.024 | -0.184 | 0.854, 0.854 | -0.032 ± 0.026 | -1.264 | 0.206, 0.412 |
| **Wing L X AD1** | -0.209 ± 0.145 | -1.436 | 0.151, 0.373 | -0.227 ± 0.205 | -1.110 | 0.267, 0.488 | -0.158 ± 0.211 | -0.748 | 0.454, 0.648 |
| **Wing L X AD2** | -0.162 ± 0.152 | -1.069 | 0.285, 0.499 | -0.374 ± 0.210 | -1.780 | 0.075, 0.246 | 0.103 ± 0.220 | 0.469 | 0.639, 0.803 |
| **Wing L X meanAD** | -0.276 ± 0.178 | -1.552 | 0.121, 0.339 | -0.452 ± 0.255 | -1.776 | 0.076, 0.246 | -0.062 ± 0.250 | -0.249 | 0.803, 0.849 |
| **Wing L X het** | -0.335 ± 0.664 | -0.504 | 0.614, 0.803 | -0.836 ± 0.906 | -0.923 | 0.356, 0.534 | 0.252 ± 0.982 | 0.257 | 0.797, 0.849 |
| **Wing P X AD1** | -0.07 ± 0.056 | -1.323 | 0.186, 0.399 | 0.012 ± 0.083 | 0.239 | 0.811, 0.849 | -0.162 ± 0.078 | -2.088 | 0.037, 0.153 |
| **Wing P X AD2** | -0.148 ± 0.057 | -2.616 | 0.009, 0.095* | -0.035 ± 0.082 | -0.433 | 0.665, 0.803 | -0.273 ± 0.078 | -3.505 | < 0.001, 0.014* |
| **Wing P X meanAD** | -0.166 ± 0.068 | -2.462 | 0.014, 0.098* | -0.022 ± 0.104 | -0.215 | 0.829, 0.849 | -0.288 ± 0.089 | -3.242 | 0.001, 0.014* |
| **Wing P X het** | -0.571 ± 0.264 | -2.162 | 0.031 , 0.145 | -0.331 ± 0.348 | -0.950 | 0.342, 0.534 | -0.948 ± 0.406 | -2.333 | 0.012 , 0.098* |

* Significant at *p* ≤ 0.10 (FDR)
