# Supplementary material for: Interactions between a Candidate Gene for Migration (ADCYAP1), Morphology and Sex Predict Spring Arrival in Blackcap Populations
Source: PLoS One. 2015 Dec 18;10(12):e0144587. doi: 10.1371/journal.pone.0144587 (PMC4684316; doi:10.1371/journal.pone.0144587)
Supplement: S5 Table — See S3 Table for details. (DOC) [file pone.0144587.s008.doc]

**S5 Table.**

|  | **All-population Analyses: All 9 Populations Set 3** | | | | | | | | |
| --- | --- | --- | --- | --- | --- | --- | --- | --- | --- |
|  | **ALL** | | | **MALE** | | | **FEMALE** | | |
|  | **Est ± SE** | **t value** | ***P, FDR P*** | **Est ± SE** | **t value** | ***P, FDR P*** | **Est ± SE** | **t value** | ***P, FDR P*** |
| **Wing L** | -1.141 ± 0.298 | -3.829 | < 0.001, 0.021* | -0.724 ± 0.404 | -1.790 | 0.073, 0.279 | -1.705 ± 0.443 | -3.846 | < 0.001, 0.021* |
| **Wing P** | -0.079 ± 0.116 | -0.679 | 0.497, 0.673 | -0.024 ± 0.155 | -0.156 | 0.876, 0.920 | -0.161 ± 0.174 | -0.924 | 0.355, 0.557 |
| **AD1** | 0.001 ± 0.004 | 0.409 | 0.683, 0.775 | -0.002 ± 0.005 | -0.429 | 0.668, 0.775 | 0.005 ± 0.005 | 1.049 | 0.294, 0.554 |
| **AD2** | -0.010 ± 0.004 | -2.620 | 0.009, 0.126 | -0.011 ± 0.005 | -2.222 | 0.026, 0.192 | -0.008 ± 0.005 | -1.473 | 0.141, 0.395 |
| **meanAD** | -0.006 ± 0.004 | -1.278 | 0.201, 0.498 | -0.010 ± 0.006 | -1.634 | 0.102, 0.306 | -0.001 ± 0.006 | -0.177 | 0.859, 0.920 |
| **het** | '-0.036 ± 0.017 | -2.148 | 0.032, 0.192 | -0.027 ± 0.023 | -1.172 | 0.241, 0.554 | -0.049 ± 0.024 | -1.991 | 0.047, 0.223 |
| **Wing L X AD1** | -0.188 ± 0.133 | -1.416 | 0.157, 0.412 | 0.180 ± 0.183 | -0.982 | 0.326, 0.554 | -0.164 ± 0.195 | -0.844 | 0.399, 0.599 |
| **Wing L X AD2** | 0.090 ± 0.143 | 0.629 | 0.529, 0.673 | -0.007 ± 0.200 | -0.037 | 0.970, 0.973 | 0.232 ± 0.205 | 1.128 | 0.259, 0.554 |
| **Wing L X meanAD** | -0.106 ± 0.164 | -0.647 | 0.517, 0.673 | -0.170 ± 0.231 | -0.733 | 0.464, 0.650 | 0.008 ± 0.232 | 0.034 | 0.973, 0.973 |
| **Wing L X het** | 0.565 ± 0.614 | 0.920 | 0.358, 0.557 | 0.817 ± 0.832 | 0.982 | 0.326, 0.554 | 0.205 ± 0.911 | 0.225 | 0.822, 0.909 |
| **Wing P X AD1** | -0.113 ± 0.058 | -1.936 | 0.053, 0.223 | -0.086 ± 0.083 | -1.035 | 0.301, 0.554 | -0.135 ± 0.082 | -1.656 | 0.098, 0.306 |
| **Wing P X AD2** | -0.060 ± 0.055 | -1.080 | 0.280, 0.554 | 0.044 ± 0.080 | 0.554 | 0.580, 0.716 | -0.169 ± 0.077 | -2.195 | 0.028, 0.192 |
| **Wing P X meanAD** | -0.133 ± 0.069 | -1.938 | 0.053, 0.223 | -0.045 ± 0.104 | -0.433 | 0.665, 0.775 | -0.207 ± 0.092 | -2.242 | 0.025, 0.192 |
| **Wing P X het** | 0.192 ± 0.247 | 0.777 | 0.437, 0.663 | 0.547 ± 0.324 | 1.687 | 0.092, 0.306 | -0.372 ± 0.382 | -0.975 | 0.330, 0.554 |

* Significant a *p* ≤ 0.10 (FDR)
