# Supplementary material for: Interactions between a Candidate Gene for Migration (ADCYAP1), Morphology and Sex Predict Spring Arrival in Blackcap Populations
Source: PLoS One. 2015 Dec 18;10(12):e0144587. doi: 10.1371/journal.pone.0144587 (PMC4684316; doi:10.1371/journal.pone.0144587)
Supplement: S6 Table — See S3 Table for details. (DOC) [file pone.0144587.s009.doc]

**S6 Table.**

|  | **All-population Analyses: All 9 Populations Set 4** | | | | | | | | |
| --- | --- | --- | --- | --- | --- | --- | --- | --- | --- |
|  | **ALL** | | | **MALE** | | | **FEMALE** | | |
|  | **Est ± SE** | **t value** | ***P, FDR P*** | **Est ± SE** | **t value** | ***P, FDR P*** | **Est ± SE** | **t value** | ***P, FDR P*** |
| **Wing L** | -0.851 ± 0.321 | -2.649 | 0.008, 0.112 | -0.599 ± 0.416 | -1.440 | 0.150, 0.350 | -1.182 ± 0.506 | -2.336 | 0.020, 0.140 |
| **Wing P** | -0.184 ± 0.121 | -1.518 | 0.129, 0.319 | -0.289 ± 0.153 | -1.881 | 0.060, 0.213 | -0.017 ± 0.197 | -0.087 | 0.930, 0.953 |
| **AD1** | 0.002 ± 0.004 | 0.649 | 0.516, 0.666 | -0.005 ± 0.005 | -1.012 | 0.311, 0.527 | 0.009 ± 0.005 | 1.850 | 0.064, 0.213 |
| **AD2** | -0.009 ± 0.004 | -2.388 | 0.017, 0.140 | -0.011 ± 0.005 | -2.234 | 0.026, 0.156 | -0.007 ± 0.006 | -1.205 | 0.228, 0.456 |
| **meanAD** | -0.004 ± 0.004 | -0.982 | 0.326, 0.527 | -0.013 ± 0.006 | -2.008 | 0.045, 0.213 | 0.003 ± 0.006 | 0.497 | 0.619, 0.743 |
| **het** | -0.034 ± 0.017 | -1.958 | 0.050, 0.213 | -0.023 ± 0.023 | -1.007 | 0.314, 0.527 | -0.051 ± 0.027 | -1.913 | 0.056, 0.213 |
| **Wing L X AD1** | -0.257 ± 0.147 | -1.747 | 0.081, 0.227 | -0.079 ± 0.199 | -0.398 | 0.690, 0.783 | -0.397 ± 0.223 | -1.781 | 0.075, 0.225 |
| **Wing L X AD2** | -0.042 ± 0.158 | -0.264 | 0.792, 0.853 | -0.086 ± 0.207 | -0.418 | 0.676, 0.783 | 0.079 ± 0.249 | 0.316 | 0.752, 0.831 |
| **Wing L X meanAD** | -0.238 ± 0.182 | -1.305 | 0.192, 0.424 | -0.129 ± 0.250 | -0.516 | 0.606, 0.743 | -0.287 ± 0.270 | -1.060 | 0.289, 0.527 |
| **Wing L X het** | 0.464 ± 0.679 | 0.683 | 0.494, 0.666 | 0.778 ± 0.870 | 0.894 | 0.371, 0.557 | 0.158 ± 1.087 | 0.145 | 0.884, 0.928 |
| **Wing P X AD1** | -0.072 ± 0.058 | -1.252 | 0.211, 0.443 | -0.058 ± 0.081 | -0.712 | 0.476, 0.666 | -0.073 ± 0.085 | -0.859 | 0.390, 0.585 |
| **Wing P X AD2** | -0.091 ± 0.058 | -1.587 | 0.112, 0.294 | 0.062 ± 0.078 | 0.798 | 0.425, 0.616 | -0.290 ± 0.086 | -3.386 | < 0.001, 0.042* |
| **Wing P X meanAD** | -0.127 ± 0.069 | -1.836 | 0.066, 0.213 | -0.002 ± 0.100 | -0.019 | 0.984, 0.984 | -0.243 ± 0.098 | -2.482 | 0.013, 0.137 |
| **Wing P X het** | -0.166 ± 0.261 | -0.639 | 0.523, 0.666 | 0.370 ± 0.320 | 1.156 | 0.248, 0.473 | -1.297 ± 0.448 | -2.894 | 0.004, 0.084* |

* Significant at *p* ≤ 0.10 (FDR)
