# Supplementary material for: Interactions between a Candidate Gene for Migration (ADCYAP1), Morphology and Sex Predict Spring Arrival in Blackcap Populations
Source: PLoS One. 2015 Dec 18;10(12):e0144587. doi: 10.1371/journal.pone.0144587 (PMC4684316; doi:10.1371/journal.pone.0144587)
Supplement: S7 Table — See S3 Table for details. (DOC) [file pone.0144587.s010.doc]

**S7 Table.**

|  | **6 Standardized Populations** | | | | | | | | |
| --- | --- | --- | --- | --- | --- | --- | --- | --- | --- |
|  | **ALL** | | | **MALE** | | | **FEMALE** | | |
|  | **Est ± SE** | **t value** | ***P, FDR P*** | **Est ± SE** | **t value** | ***P, FDR P*** | **Est ± SE** | **t value** | ***P, FDR P*** |
| **Wing L** | -1.050 ± 0.340 | -3.086 | 0.002, 0.028* | -0.754 ± 0.466 | -1.618 | 0.106, 0.324 | -0.754 ± 0.499 | -2.970 | 0.003, 0.032* |
| **Wing P** | -0.256 ± 0.127 | -2.020 | 0.043, 0.226 | -0.220 ± 0.171 | -1.283 | 0.200, 0.525 | -0.315 ± 0.189 | -1.670 | 0.095, 0.324 |
| **AD1** | 0.003 ± 0.004 | 0.779 | 0.436, 0.684 | -0.002 ± 0.006 | -0.400 | 0.689, 0.839 | 0.009 ± 0.005 | 1.606 | 0.108, 0.324 |
| **AD2** | -0.010 ± 0.004 | -2.470 | 0.014, 0.118 | -0.010 ± 0.006 | -1.725 | 0.085, 0.324 | -0.011 ± 0.006 | -1.838 | 0.066, 0.277 |
| **meanAD** | -0.005 ± 0.005 | -1.018 | 0.309, 0.590 | -0.010 ± 0.007 | -1.359 | 0.174, 0.487 | -0.0003 ± 0.007 | -0.061 | 0.952, 0.952 |
| **het** | -0.033 ± 0.018 | -1.842 | 0.066, 0.227 | -0.018 ± 0.026 | -0.704 | 0.481, 0.722 | -0.052 ± 0.025 | -2.082 | 0.037, 0.222 |
| **Wing L X AD1** | -0.093 ± 0.174 | -0.536 | 0.592, 0.777 | 0.148 ± 0.256 | 0.578 | 0.563, 0.777 | -0.289 ± 0.234 | -1.234 | 0.217, 0.536 |
| **Wing L X AD2** | -0.015 ± 0.155 | -0.095 | 0.924, 0.947 | 0.022 ± 0.211 | 0.106 | 0.916, 0.947 | -0.046 ± 0.230 | -0.199 | 0.842, 0.907 |
| **Wing L X meanAD** | -0.076 ± 0.196 | -0.387 | 0.699, 0.839 | 0.097 ± 0.284 | 0.343 | 0.731, 0.853 | -0.209 ± 0.269 | -0.779 | 0.436, 0.684 |
| **Wing L X het** | -0.385 ± 0.697 | -0.553 | 0.581, 0.777 | -0.234 ± 0.949 | -0.247 | 0.805, 0.890 | -0.648 ± 1.027 | -0.631 | 0.528, 0.765 |
| **Wing P X AD1** | -0.031 ± 0.062 | -0.501 | 0.616, 0.784 | 0.0246 ± 0.089 | 0.277 | 0.781, 0.887 | -0.072 ± 0.090 | -0.805 | 0.421, 0.684 |
| **Wing P X AD2** | -0.062 ± 0.058 | -1.078 | 0.281, 0.580 | 0.094 ± 0.083 | 1.122 | 0.262, 0.579 | -0.244 ± 0.079 | -3.085 | 0.002, 0.028* |
| **Wing P X meanAD** | -0.076 ± 0.072 | -1.058 | 0.290, 0.580 | 0.085 ± 0.109 | 0.773 | 0.440, 0.684 | -0.216 ± 0.096 | -2.247 | 0.025, 0.175 |
| **Wing P X het** | -0.313 ± 0.272 | -1.149 | 0.251, 0.579 | 0.327 ± 0.364 | 0.898 | 0.369, 0.674 | -1.336 ± 0.403 | -3.315 | < 0.001, 0.028* |

* Significant at *p* ≤ 0.10 (FDR)
